# Mental suffering in family daily life: a temporal journey according to Merleau-Ponty

**DOI:** 10.1590/0034-7167-2023-0258

**Published:** 2023-11-27

**Authors:** Patricia Anjos Lima de Carvalho, Vanessa Thamyris Carvalho dos Santos, Marlene Gomes Terra, Márcia Aparecida Ferreira de Oliveira, Ricardo Henrique Soares, Edite Lago da Silva Sena

**Affiliations:** I Universidade Estadual do Sudoeste da Bahia. Jequié, Bahia, Brazil; II Universidade Federal de Santa Maria. Santa Maria, Rio Grande do Sul, Brazil; III Universidade de São Paulo. São Paulo, São Paulo, Brazil

**Keywords:** Mental Health, Family Relations, Philosophy, Nursing, Community Mental Health Services, Salud Mental, Relaciones Familiares, Filosofía, Enfermería, Servicios Comunitarios de Salud Mental, Saúde Mental, Relações Familiares, Filosofia, Enfermagem, Serviços Comunitários de Saúde Mental.

## Abstract

**Objectives:**

to describe the family’s experience in relation to daily life with a family member experiencing mental suffering.

**Methods:**

a qualitative, descriptive, phenomenological study grounded in Merleau-Ponty’s ontology of experience was conducted in ten households in a city in the state of Bahia, Brazil, where 24 participants of the Intersubjectivity Wheels reside. The descriptions produced were subjected to the Ambiguity Analytics technique.

**Results:**

the descriptions were categorized into: absence as a creative power of the sense of “being” and “not being a family”; and exclusion and acceptance as expressions of mental suffering in the family context.

**Final Considerations:**

the experience of mental suffering in the family’s daily life is marked by ambiguous feelings, such as joy and sadness, disappointment and satisfaction, lack of love and love. However, experiencing these feelings can mobilize the desire to “become” a family, increase the sense of autonomy and independence, and drive the formation of new family configurations.

## RESUMO

**Objetivos:**

descrever a experiência da família em relação ao cotidiano com o familiar em sofrimento mental.

**Métodos:**

estudo qualitativo, descritivo, do tipo fenomenológico, fundamentado na ontologia da experiência de Merleau-Ponty, realizado em dez domicílios de uma cidade do estado da Bahia (Brasil), onde residem os 24 participantes das Rodas de Intersubjetividade. As descrições produzidas foram submetidas à técnica Analítica da Ambiguidade.

**Resultados:**

as descrições foram categorizadas em: ausência como potência criadora do sentido de “ser” e “não ser família”; e exclusão e acolhimento como expressão do sofrimento mental no contexto da família.

**Considerações Finais:**

a experiência do sofrimento mental no cotidiano da família é marcada por sentimentos ambíguos, como alegria e tristeza, decepção e satisfação, desamor e amor. Entretanto, a vivência desses sentimentos pode mobilizar o desejo de “tornar-se” família, aumentar o senso de autonomia e independência, e impulsionar a formação de novas configurações familiares.

## RESUMEN

**Objetivos:**

describir la experiencia de la familia en relación con la vida diaria de un miembro familiar que experimenta sufrimiento mental.

**Métodos:**

se realizó un estudio cualitativo, descriptivo y fenomenológico basado en la ontología de la experiencia de Merleau-Ponty en diez hogares de una ciudad en el estado de Bahía, Brasil, donde residen 24 participantes de los Círculos de Intersubjetividad. Las descripciones producidas fueron sometidas a la técnica de Análisis de Ambigüedad.

**Resultados:**

las descripciones se clasificaron en: la ausencia como un poder creativo del sentido de “ser” y “no ser una familia”; y la exclusión y aceptación como expresiones del sufrimiento mental en el contexto familiar.

**Consideraciones Finales:**

la experiencia del sufrimiento mental en la vida diaria de la familia está marcada por sentimientos ambiguos, como alegría y tristeza, decepción y satisfacción, falta de amor y amor. Sin embargo, experimentar estos sentimientos puede movilizar el deseo de “convertirse” en una familia, aumentar el sentido de autonomía e independencia, y promover la formación de nuevas configuraciones familiares.

## INTRODUCTION

The appropriation of madness by psychiatric knowledge, which made it manageable, medicalized, and labeled it as a disease, resulted in the exclusion of the mentally ill from social and family circles^(1-4)^. The isolation in asylums was justified ambiguously, as psychiatry sometimes blamed the family for the illness and other times portrayed them as victims^(2-5)^. The family’s relationship with the ‘mad’ individual was limited to guiding them to the psychiatric hospital for medical care, as well as visiting and providing information about their history^(2)^.

The proposals for deinstitutionalization, driven by the Psychiatric Reform movement in Brazil in the 1970s, constitute a project to replace psychiatric practices with community-based care involving families^(1,4,6)^. Thus, the psychosocial care model started to guide the sharing of care protagonism between users and family members, aiming at social reintegration in terms of complexity and existence^(5,7)^.

This care model demands the creation of a Psychosocial Care Network that offers support to families to recognize emerging daily changes involving different configurations and dynamics and adapt to them^(8)^. These changes occur in individual and family life cycles and are part of human development, within the context of political, cultural, demographic transformations, among others, which somehow impact families^(7-10)^.

Family involvement in care becomes more evident when one of its members experiences suffering that requires the group’s reorganization to deal with the new situation and relies on the family’s potential to reframe the past, present, and future, thus fostering solidarity among its members^(11-12)^. In this understanding, care encompasses ways of being grounded in values, attitudes, empathy, and affections that enable people to care for others while caring for themselves^(12)^.

As a result of transformations and needs felt by families experiencing mental suffering, various social actors have tried to support them, from religious leaders, physicians, educators, to more recently, family therapists, all seeking to build alliances and positive encounters^(3)^. Among healthcare professionals, nursing professionals stand out, who, through consultations, group activities, home visits, and other actions, have sought to develop therapeutic relationships based on listening, dialogue, and meeting the needs revealed through intersubjectivity built with families^(6-7,13-14)^.

In this context, the family can be considered both an extension of the family member experiencing mental suffering and an informal support network for them, and their participation in care can strengthen emotional bonds and assist in their social relationships^(7,9)^. This understanding has motivated healthcare professionals to develop practices aimed at recognizing and valuing everyday situations that mobilize care in the community through initiatives of continuous education, allowing the construction of knowledge, autonomy, empowerment, and social reintegration^(7,15-16)^.

Although the consulted authors^(6-7,9,13-14)^ agree on the importance of family care actions, discussions and studies that systematize contextualized knowledge regarding the family’s perception of how daily instability and unpredictability interfere with the health and suffering of its members were not found. The experience in the field of mental health has allowed for closer engagement with families experiencing mental suffering and provided a space for listening to the experience of suffering in their daily lives, as an unfolding of time, a current experience intertwined with an imposing past, but not remaining as an eternal present.

The study’s theme is essential, considering that the potential for (de)institutionalization by families experiencing suffering in their daily lives can shed lighter on the phenomenon, as the family can be a conducive setting for the construction of autonomy, empowerment, and social reintegration.

This reflection has allowed us to understand that, just as daily life consists of dynamic events, experiences of mental suffering can also manifest themselves dynamically and ambiguously, both within the same family and among different families.

## OBJECTIVES

To describe the experience of families in their daily life with a family member experiencing mental suffering.

## METHODS

### Ethical aspects

The research adhered to the ethical criteria established by Resolution No. 466/2012 of the National Health Council and was approved by the Research Ethics Committee of the State University of Southwest Bahia (UESB). To ensure anonymity, participants from the Psychosocial Care Center II (PCC II) chose codenames of birds, while other family members chose names of animals. The recording of experiential descriptions was authorized by participants through the signing of the Informed Consent Form (ICF).

### Theoretical-methodological framework

The study employed the phenomenological framework of Maurice Merleau-Ponty, which addresses temporality and introduces the notion that consciousness organizes itself in relation to the flow of time^(17)^. This approach was developed based on the understanding that the past, as a lack of self-knowledge (habitual body), emerges supported by the present experience (current body) as a projection into the future (perceptive body), an openness to becoming^(17-18)^. According to Merleau-Ponty, this simultaneity appears in the contact of the perceptive subject with the world, “as if the visibility that animates the sensible world emigrated not outside the body but to another body, lighter, more transparent, as if it changed from flesh, abandoning that of the body for that of language”^(17)^.

### Study design

Descriptive research with a qualitative phenomenological approach. The Consolidated Criteria for Reporting Qualitative Research (COREQ) was used to guide the methodology.

### Methodological procedures

#### Study setting

The study was conducted from July 2017 to June 2018 in a city in the state of Bahia, Brazil, in the households of 24 participants who were part of 10 families of PCC II users. The procedure for selecting families took place in a meeting held at the service, where thirteen users expressed interest and indicated other members of their families to participate in the research. However, three users decided not to participate.

#### Data source

The study involved 10 families, with 24 family members participating in the experiential productions related to their daily life with mental suffering. The selection criteria for family members were as follows: being over eighteen years old; being a user of PCC II or a family member (only families with at least one member registered in the service could participate); and being a significant person for the family, even if not related by blood. Regarding the characteristics of the interviewees, the majority earn a minimum wage, have completed elementary education, are of the Protestant religion, and are between 18 and 77 years old.

#### Data Collection and Organization

The experiential descriptions were produced through twenty-four phenomenological interviews, ten Intersubjectivity Wheels held in households, and one Intersubjectivity Wheel conducted at UESB. The first author of this text always accompanied by one of the co-authors guided these sessions. In this article, we present some descriptions that emerged from the dialogue during the intersubjectivity wheels. The guiding reflection for these sessions was to contrast the asylum model, where a person in crisis would be subject to hospitalization, with the psychosocial model, which advocates for treatment in the community, integrating various resources and relationships with significant individuals. The psychiatric hospital is seen as the last resort in this approach.

When developing the strategy of intersubjectivity wheels, we drew inspiration from conversational circles, which serve as spaces for transformation, encouraging open and dialogical discussions^(19)^. These discussions involve listening and speaking among various interlocutors who construct perceptions through interaction with others^(20)^. The goal is to align with the philosophical theoretical reference and understand experiences through a “return to the things themselves”, reconnecting with the intuitively lived world^(18)^.

The intersubjectivity wheels commenced by presenting a text that portrayed the territory where the families live, based on the experiences described by each participant during the phenomenological interviews. We used the term “Clinic” to refer to the notion that considers the wandering of people with mental suffering through the territory as an opportunity to develop clinical experiences involving the discovery of resources that contribute to social reintegration^(21)^.

#### Work Stages

The intersubjectivity wheels were conducted in the following stages: presentation of the research project, welcoming and integration between researchers and families during a meeting at PCC II (acclimatization); reading of the text containing the portrayal of the territory; construction of the “I can” map (a method of presenting the social spaces that appeared in the peripatetic synthesis as illustrative figures on cardboard as a single image); network game (a playful approach to lead the family in reflecting on possibilities of integration within the territory, using atomic markers to connect the figures on the “I can” map and justifying the choices); closing (evaluation of the activity).

#### Data Analysis

The phenomenological interviews and intersubjectivity wheels were concluded when the essence of the phenomenon was revealed. The transcriptions of the texts resulting from the intersubjectivity wheels underwent Ambiguity Analytics, a strategy to understand experiential descriptions originating from phenomenological-based studies. This approach involves reading the empirical material meticulously to perceive the figure-ground relationship that emerges from the text and its subtexts. The analysis involves identifying the theses supporting the objectification of things as they are and perceiving expressions that reveal ambiguities and characterize profiles of a whole. Subsequently, the data are categorized^(22)^. The families participating in an intersubjectivity wheel at UESB presented and validated the categories.

## RESULTS

From the analysis, we observed the ambiguities present in the experience of families living with mental suffering in their daily lives, as revealed in this article through two categories: absence as a creative power of the sense of “being” and “not being” a family; and exclusion and acceptance as expressions of mental suffering in the family context.

### Absence as a Creative Power of the Sense of Being and Not Being a Family

In this category, we present descriptions from study participants that highlight the impact of family absence in daily life, indicating a desire for belonging, affection, and structure, all driven by the perception of “being” and “not being” a family in the context of mental suffering.

The absence of the family was intertwined with feelings of loneliness, sadness, and disappointment, which, on one hand, reveal the longing for affection, companionship, and attention, and on the other hand, can foster independence and autonomy:

[...] *I’ve always lived alone.* [...] *I had my stepmother and my sister at home, but none of them interfered in my life. I never had that family support; my father was always traveling. I learned to live alone, and that wasn’t entirely my fault because I never had the structure I had when I lived with my mother; she provided structure, but then she left. The blame today is on my father; the person I am now is thanks to my past. I never had a real father; he was just a provider, only giving advice from a distance, over the phone.* (Pitbull)

[...] *no one can live alone.* (Ovelha)

[...] *it’s not that simple! There are two sides.* [...] *I went through the experience of marriage, and when I see myself living alone, sometimes I feel very happy. I know it’s not healthy to live alone. There are also bad moments, moments of sadness.* (Fênix)

[...] *I also live alone* [laughs]*, and I really enjoy my independence, it’s great, it’s everything.* [...] *You’re not enjoying it because it’s new; you want to have a partner. But in my case, no.* (Tigre)

The descriptions reveal ambiguities related to the absence of support from the family, particularly from the father or spouse of the family member experiencing mental suffering. However, they also seem to open up perspectives for more fulfilling experiences in the future.

[...] *I can improve, but the person who was raised this way will never change.* [...] *it all comes back to the pillar of family* [...]*, I know a person can live without one, but it’s difficult. We can live without a father, but it’s difficult.* (Pitbull)

[...] *It’s true, because I also didn’t have my father, when my grandmother died, I had to look up to others as examples to become who I am* [...]. (Avestruz)

[...] *I took it as an experience that if I have a son or daughter, I’ll do things differently: I won’t cheat on my wife, won’t have more than one wife; I’ll take care of my child; I’ll be present in my family.* (Pitbull)

[...] *my son liked to play soccer, and I played with him too* [...]. (Arara)

[...] *I always liked playing soccer with the boys, but he always prevented me from doing that* [...] *I would jump the fence, find a way to go.* (Ursa)

[...] *what we experienced in the past along with what we’re living now, taking all of that and projecting it into the future. It just takes willpower.* [...] *who knows, we might get lucky in life, there could be a turnaround!* (Arara)

The descriptions showed that, even though the family members reside in the same city, there is not enough closeness to actively engage the family in supporting the PCC user, despite expressing a desire to do so:

[...] *it hurts not to have a father, not to have a mother,* [...] *but she* [the girlfriend] *came, she filled my loneliness* [...] *the only thing missing now is to spend more time with my mother* [a PCC user] [...]. (Pitbull)

[...] *his life is just about the house, social media, girlfriend, and work, now that I’m not there.* (Avestruz)

[...] *my aunt spoke to my sister to take care of me, but she* [my sister] *said I wasn’t sick. When she saw that I was going to kill her daughter because I went crazy, then she had me hospitalized* [...] [even now]*, my brothers would take care of me, but not her.* (Galinha)

[...] *she has the financial means, she could help, but she doesn’t.* (Rottweiler)

### Exclusion and Acceptance as Expressions of Mental Suffering in the Family Context

In this category, descriptions appear that reflect, on one hand, the exclusion of the consanguineous family, intertwined with the experience of mental suffering and the individualism of its members; and, on the other hand, the emergence of a new family configuration through the coexistence of feelings such as respect, friendship, and joy among PCC users.

The descriptions reveal the exclusion within the family, demonstrated through feelings of disinterest, disunity, lack of love, ways of expressing distance, and the lack of listening and acceptance that challenges the notion that there is always love within the family.

[...] *we drifted apart; no one sits down to ask what’s going on, no one wants to know. We’re too individualistic.* [...] *I was surprised to hear that you, felt excluded by the family.* (Tigre)

*There are many exceptions, but I felt it, I really did. I also said that after my own marriage; when I got sick, we separated because of the illness* [...]. (Fênix)

[...] *her duty* [the wife’s duty] *wasn’t to leave, it was to help.* (Ovelha)

[...] *I think there’s disunity among the siblings* [...] *each one wants to live their own life, doesn’t care about the other,* [...] *I think, on the part of us, the children, it’s a lack of affection, each one lives their own life, cares, but not as they should. As for me, the reconstruction of our relationship has already started; he knows that, it’s been a long time.* (Ursa)

[...] *kids only care about their father if he has money. I don’t have that. They might think I have problems, that I’m just lying around alone, like a dog, and think: I’ll go there and help him with something. I didn’t want anything, just affection and love. It’s easier for you to come here* [to my home]*, take me somewhere, than for them* [the family]*; that’s why I go out alone, travel all over, alone.* [...]*. These people want us to die, when someone loves another, you know it.* (Arara)

On the other hand, the families revealed the potential of dialogue that occurs in PCC for the transcendence of more fulfilling feelings, such as acceptance, trust, joy, friendship, and belonging, which create new relationships and a new family configuration, as shown in the descriptions:

[...] *it makes me sad because I can’t go there anymore* [to the city where I lived before the crisis]*; the family was angry with me because he* [my husband] *died more because of the stab I gave him, it hit his heart. So, the family was upset with me.* [...] *I like to talk to my colleagues to forget the past because it’s still in my head.* [At PCC] *I feel happy; it’s like I was born again there; we all speak the same language, I open up, talk about everything, there are so many people I like to chat with, it’s like a family.* (Galinha)

[...] *when I’m feeling a bit down during the week, I talk to Bambu, and then I leave the PCC II feeling renewed. It’s an essential place, a safe harbor; the PCC is like that* [...]. (Fênix)

[...] *when you’re with someone you like, you won’t even notice the crowd around you.* [...] *talking about different things, that’s wonderful.* (Tigre)

[...] *One day, Cisne said: Arara, we can’t be angry because we’re friends. So, I said: but you insulted my mother* [...]*, I was going to leave PCC because of you, and he said: no, don’t leave, captain.* (Arara)

[...] *he encourages other people to live in society because they see the example there, one helping the other.* (Ursa)

[...] *That’s why I say they are my family; day by day, we’re together, we’re a family.* (Arara)

## DISCUSSION

Looking at the experience of mental suffering in the daily life of families with PCC II service users, in light of the notion of temporality as inherent intentionality, allows us to understand the temporal activity of the family world as a whirlwind^(17)^. This term, used by the author, refers particularly to the experience of intercorporeality that occurs in relationships between individuals. Within this whirlwind, there is a process of temporal self-constitution and, therefore, differentiation of being in the world through the experience of transcendence between individuals, whose identity is made and remade in relation to each other^(17-18)^.

Temporal constitution occurs through the constant updating of the present, which carries within itself a horizon of the past, referring to past sociohistorical experiences and intuitions, and a horizon of the future, characterized as prospective virtuality filled with desires and hopes^(17)^. According to Merleau-Ponty, “the past adheres to the present, and not the consciousness of the past adhering to the consciousness of the present”^(17)^.

The experience of mental suffering in the daily life of the family constitutes a phenomenal field in which the temporal flows of its members intertwine, and various profiles emerge, bringing with them ambiguities. Among these profiles, the feeling of loneliness due to the distancing of family members reveals modes of “being” and “not being” a family, which are updated in the simultaneity of dimensions of presence and absence, visibility and invisibility. These profiles are shown to be essential features of both perceived and intersubjective experiences.

The invitation to the family to turn their gaze to the experience of mental suffering revealed the absence, both as “not being” a family and as the desire to “become” a family in everyday life. It demonstrated the participants’ perception that the family does not adequately accompany the member with mental suffering in their daily life. It also revealed a horizon of coexistence that allowed families to recognize the lack of affection and individualism, rather than listening and understanding their experiential dynamics^(9,23)^.

This perception of the lack of affection and individualism, which permeates the relationships between the family and the member with mental suffering, is related to the notion of the ephemeral nature of relationships that seem subject to the logic of the market. Relationships established with others, which should be enduring, are treated as commodities and products that can be replaced at any time^(23)^.

From this perspective, affection and a sense of belonging in a relationship or group would be replaced by weakened and dehumanized relationships, which are, however, more functional in the logic of utilitarian culture^(23)^. This understanding reverberated in the participants’ descriptions when attempting to deconstruct Galinha’s thesis that “there is always love in the relationship between parents and children”. They did so by expressing ambiguous feelings related to the lack of attention and affection, as seen in the example of Arara, who states that she does not have money to give to her children, and Pitbull, who says he never had a present father, only a father focused on monetization.

The descriptions also reveal ambiguities related to the feeling of loneliness. The experience of loneliness sometimes appears as a promoter of independence, autonomy, and empowerment in life, while other times, it coexists with feelings of sadness and joy, a desire to continue living alone, and a desire for companionship. This ambiguous movement of perception supports the suspension of the thesis present in the descriptions that “nobody can live alone”. The desire for companionship became more evident in the descriptions of PCC II users, Fênix, and Avestruz, corroborating a study that highlights the need for care and protection in the face of the intense journey they commonly go through^(24)^.

However, Pitbull’s description, “I never had a family”, revealed a return to his sensitive dimension, that is, a return to the things themselves, bringing to the surface feelings of abandonment and contempt experienced in his relationship with his sister and stepmother. This understanding represents the absence of the family as a “non-being”, an invisible entity, which, on the one hand, shows the distancing from the family constituted by his father when he married another woman^(8,25)^, but on the other hand, reveals the sense of being a family, based on the desire to build relationships of respect, trust, belonging, structure, and affection.

Thus, the “sensitive flesh”, described by Merleau-Ponty^(17-18)^ as language, the experience of speech, reflection, opens up to new and endless transformations through the communication of more gratifying feelings, such as affection, care, and solidarity. These aspects reveal the coexistence with others, a generality that mobilizes the transformation of personhood and cultural being.

The identification with a gratifying experience brings about such a radical change in perception (the “body”) that we no longer perceive where and when it all began. In other words, while the universality of feeling (the feeling of coexistence) mobilizes us towards alterity, towards establishing new identifications as historical beings, it also produces new coexistences and generalizations^(17-18)^.

This understanding indicates the participants’ readiness for the transcendence towards more gratifying feelings in the future, as observed in Pitbull’s description where he reveals his intention to build an idealized nuclear/traditional family^(10,26)^. While he deconstructs the thesis that “a person structured in a certain way cannot change”, he reaffirms patterns present in behaviors transmitted through generations^(10)^.

Pitbull’s expression of the notion of family reveals a way of being a family characterized by differentiation and interweaving. This supports the study that highlights the role of the family in society as a generative relational identity involving interpersonal recognition, including the “natural” structure as a process of generation, transmission, and expectation of cultural transformation^(27)^. While the descriptions show the desire to redefine family life, they also demonstrate the need for emotional involvement and becoming a source of hope, happiness, solidarity, and creativity^(12,26)^.

Similarly, the participants’ perception of exclusion and inclusion in the family context is not simply an assimilation of silent linguistic meanings but rather a diacritical structure that provided a set of deviations, intervals, and discontinuities between the sensitive components of the perceived objects, which were revealed ambiguously^(17-18)^. At times, it appears as abandonment, loneliness, and absence from the consanguineous family, and at other times, it appears as acceptance, friendship, and presence from other users and PCC professionals, who became like a family to the family members experiencing mental suffering.

The intersubjectivity with the participants enabled the observation of a way of being a family in which, despite consanguinity, feelings of distance, disinterest, exclusion, disunity, lack of love, and a lack of listening and acceptance coexist. These ambiguous experiences deconstruct the thesis that “there is always love between family members”. However, studies emphasize that the process of identification with the family suggests the recognition of the love that exists among its members, assuming that one loves when they recognize the other’s need to love and be loved^(12,23)^. This understanding acknowledges the power of love to become a generalized symbolic means of exchange between the family and society as a whole^(23)^.

By revealing the potential of dialogue between family members and other PCC II users, families made the experience of love intertwined with gratifying feelings, such as acceptance, respect, trust, joy, and friendship, evident. These aspects demonstrate both caring relationships and the constitution of a new family configuration, one that strengthens the sense of belonging, of being among equals^(23)^.

The immersion in the participants’ experiential context enabled an understanding that dialogue about the experience of mental suffering in the family’s daily life exists between “two silences”: one of “expression of the mute experience, ignorant of its own meaning”, but which can break our contact with things and “take us out of a state of confusion” in which we found ourselves with all things, to “awaken us to the truth of their presence, to make their relief and our connection with them sensitive”; and the other of language, as “the enigma of being, which, beyond the movement of pure meanings, appears in the silent mass of speech, that which is not of the order of the sayable”^(17)^.

The intersubjectivity built in the study demonstrated the need to approach families experiencing mental suffering with sensitivity, in order to foster dialogues, connections, and open spaces for the redefinition of family ways of being. This is essential to recognize that ambiguities such as sadness and joy, love and hatred, pride and disappointment are inherent to human nature and, therefore, are part of the family’s daily life.

Therefore, this approach to families reveals the need to mobilize the sensitivity of nurses and other mental health professionals regarding the desire to build creative and supportive paths to work within the family context, which experiences mental suffering as a return to the things themselves.

This sensitivity of health professionals is crucial in dealing with ambiguities that family members may evoke in the everyday service, as the family can sometimes be perceived as co-responsible for the care, while at other times, they may be seen as needing care themselves. The ambiguity between caring and being cared for seems to pose a challenge for health professionals, as the family member who cares also needs care^(28)^.

The family is also perceived by the healthcare team as essential in the process of psychosocial rehabilitation, as the paradigm of mental health care aims to share responsibilities for care actions^(28)^.

For the family to be considered the foundation of mental health care, it is necessary to recognize the problems and difficulties specific to living with individuals experiencing psychological suffering. It is well known how challenging this coexistence can be for families, as it involves understanding and dealing with certain behaviors (talking to oneself, disrupting sleep cycles, social withdrawal, mood changes, neglect of personal hygiene), which can evoke ambiguous feelings such as love, shame, and anxiety. It is essential for health professionals to be attentive to the signs that can lead to family overload, offering support and guidance in this regard^(29)^.

Families can play a crucial role in the success of treatment, provided they receive guidance and support from health service professionals^(29)^. The potential of the family to become an existential device for care is intertwined with its willingness to build and rebuild virtues. Families experiencing mental suffering can bring visibility to these virtues, which can be understood as relational assets intertwined with feelings of happiness within the family context, such as reciprocity, generosity, and solidarity. These aspects present possibilities for the person experiencing mental suffering to (re)conquer family ties^(30)^.

Thus, through dialogue and intersubjectivity, health professionals can learn about what exists, what is experienced, and what is lived by families, in order to understand their singularities and daily demands and, through common language, express ways of caring in completeness that construct and are constructed as intersubjectivity.

### Study limitations

This research should be seen considering the limitation of privileging the speech of family members present in their homes during the moments of intersubjectivity circles, which means that some participants from phenomenological interviews were not heard in the collective context. Considering that the participants revealed the potential of affective relationships developed in PCC II for the construction of new family configurations, it is suggested to conduct new studies that aim to listen to other social actors in different points of attention.

### Contributions to the field of nursing and health

The study’s main contribution to theoretical and scientific knowledge is the recognition of the potency of dialogicity to “return to the things themselves”, that is, to family experiences, which can be seen as producers of care for families in the field of mental health. In this sense, the work in this field, especially when aiming to involve the family in care, requires a disposition from the nurse and other workers in the area to place themselves at the same time as learners and mediators of empowerment, which can help the family experiencing mental suffering to become autonomous and creative in their way of deinstitutionalization.

## FINAL CONSIDERATIONS

The study sheds light on the experience of the family living with mental suffering in their daily life, which is permeated by feelings of loneliness, sadness, and disappointment, evidenced in the expression of “not being” a family, intertwined with the desire to “become” a family. At the same time, the descriptions reveal the flip side of “not being” a family as a daily absence, which both generates the search for new approaches and dialogues and enhances the sense of autonomy and independence, thus opening perspectives for more rewarding future experiences.

However, the study reveals the fragilities of family bonds in the daily life of living with mental suffering, evidenced as a lack of listening, a reduction in the sense of belonging, and even the exclusion of the ‘mentally ill’ from their own consanguineous family. It also showed the family’s openness, especially of the person experiencing mental suffering, to build connections with other users and workers of PCC, which inscribes this approach as a new family configuration in the field of mental health.

The reflection built in intersubjectivity with the study participants, in an atmosphere of freedom, autonomy, and pluralism of ideas, which would not be known if they did not possess a body and sensitivity, constitutes the opening to a new perspective on the experience of the family living with mental suffering. This understanding is based on a philosophical style of being a professional in the field of mental health, which uses a dialogical relationship with the subjects within their territory to build possibilities of care for the family.
